# Efficiency of Peptide Nucleic Acid-Directed PCR Clamping and Its Application in the Investigation of Natural Diets of the Japanese Eel Leptocephali

**DOI:** 10.1371/journal.pone.0025715

**Published:** 2011-11-01

**Authors:** Takeshi Terahara, Seinen Chow, Hiroaki Kurogi, Sun-Hee Lee, Katsumi Tsukamoto, Noritaka Mochioka, Hideki Tanaka, Haruko Takeyama

**Affiliations:** 1 Department of Life Science and Medical Bioscience, Waseda University, Shinjuku-ku, Tokyo, Japan; 2 National Research Institute of Aquaculture, Yokosuka, Japan; 3 Dong-A University, Saha-gu, Busan, Korea; 4 Atmosphere and Ocean Research Institute, The University of Tokyo, Chiba, Japan; 5 Kyushu University, Fukuoka, Japan; 6 National Research Institute of Aquaculture, Mie, Japan; University of New South Wales, Australia

## Abstract

Polymerase chain reaction (PCR)-clamping using blocking primer and DNA-analogs, such as peptide nucleotide acid (PNA), may be used to selectively amplify target DNA for molecular diet analysis. We investigated PCR-clamping efficiency by studying PNA position and mismatch with complementary DNA by designing PNAs at five different positions on the nuclear rDNA internal transcribed spacer 1 of the Japanese eel *Anguilla japonica* in association with intra-specific nucleotide substitutions. All five PNAs were observed to efficiently inhibit amplification of a fully complementary DNA template. One mismatch between PNA and template DNA inhibited amplification of the template DNA, while two or more mismatches did not. DNA samples extracted from dorsal muscle and intestine of eight wild-caught leptochephalus larvae were subjected to this analysis, followed by cloning, nucleotide sequence analysis, and database homology search. Among 12 sequence types obtained from the intestine sample, six were identified as fungi. No sequence similarities were found in the database for the remaining six types, which were not related to one another. These results, in conjunction with our laboratory observations on larval feeding, suggest that eel leptocephali may not be dependent upon living plankton for their food source.

## Introduction

DNA-based analysis has become a popular tool for molecular diet analysis [1], [2], [3], [4], [5], [6]. An orthologous DNA region from a wide variety of organisms can be amplified by polymerase chain reaction (PCR) with universal primers, which are subsequently subjected to nucleotide sequence analysis followed by homology search. Because PCR usually favors amplification of dominant DNA molecules, accurate molecular diet analysis is difficult due to host organism contamination. For example, when stomach content and host tissue cannot be well separated, as in the case of an invertebrate or their larvae, the crude DNA preparations may contain a substantial amount of host DNA. Attempts to determine prey organisms of the phyllosoma larvae of spiny and scyllarid lobsters were performed [2], [4], in which 18S rDNA molecules were amplified using a crude DNA template extracted from the hepatopancreas, cloned, and subjected to restriction fragment length polymorphism analysis to select clones showing non-host restriction patterns. They found nearly 90% of the 2,341 clones examined were of the host lobster larvae, indicating that a substantial amount of contamination from the host genome occurred.

PCR-clamping using blocking primer and DNA-analogs, such as peptide nucleotide acid (PNA) and locked nucleotide acid, may be a promising technique to inhibit amplification of excess non-target DNA [5], [6], [7]. Using universal primers to amplify ribosomal DNA internal transcribed spacer 1 (ITS1) with spiny lobster (the genus *Panulirus*) specific PNA, a remarkable PCR-clamping of the host DNA and detection efficiency of the prey DNA was demonstrated [6]. However, the positional relationship between PNA and PCR primers, and mismatch between PNA and template DNA in relation to the clamping efficiency, was not further investigated.

We have applied the PCR-clamping technique using PNA to investigate the natural diet of leptocephalus larvae of the Japanese eel, *Anguilla japonica*. The natural diet of the eel leptocephali has been a mystery [8] and this information may prove important for developing better artificial food in aquaculture. To design eel-specific PNA, we determined the ITS1 sequence of the Japanese eel and found several intra- and inter-individual nucleotide substitutions. In this study, using the eel ITS1 variants, we investigated the effects of PNA position and mismatch between PNA probes and the target sequence. We also report results of our preliminary attempt to investigate the natural diet of eel leptocephali using PCR clamping.

## Methods

### Ethics statement

All animal sampling in this study complied with the Council of the European Communities Directive 86/609/EEC regarding the protection of animals used for experimental and other scientific purposes, and fully complied with local fisheries management and marine protected area controls. Wild adult and larval eels captured with trawl nets deployed from research vessels were dead on retrieval and sampled at that time.

### Samples and DNA extraction

Four adults (eel00, eel01, eel02, and eel03) of the Japanese eel (*Anguilla japonica*) were used in this study; two (eel00 and eel01) were cultured individuals and the others (eel02 and eel03) were caught in the southern part of the West Mariana Ridge [9]. These adult samples were used to investigate intra-specific variation in the ITS1 region. Early stage leptocephalus larvae (c.a. 10–17 mm total length) of the Japanese eel were caught in 2009 and 2010 by RV Kaiyo Maru and Shoyo Maru (Fisheries Agency, Japan) using an Isaacs-Kidd Midwater Trawl net at the southern part of the West Mariana Ridge. Four larvae (designated as 163, 165, 167 and 177) in 2009 and four (designated as St23-10, St23-11, St24-12 and St30-15) in 2010 were rinsed with sterile sea water on board, fixed in 70% ethanol, and transferred to the laboratory. Prior to dissection in the laboratory, the body surface of the leptocephali was rinsed with 70% ethanol. The dorsal muscle of the larvae was first dissected using sterile forceps and used as a control. Subsequently, the intestine was dissected and used for diet analysis. Crude DNA was extracted from these adult and larval samples using DNeasy Blood and Tissue Kits (Qiagen, Tokyo, Japan). A DNA sample of the Japanese spiny lobster (*Panulirus japonicus*) derived from our laboratory stock was used as a model prey organism.

### Sequence analysis

Nucleotide sequences of the PCR primers used are shown in Table 1. SP-1-5 and 5.8S were used to amplify the long fragment of one adult (eel00). ITS1 of the other adults was amplified using ITS5 and 5.8S. PCR was performed in a reaction mixture (total 25 µl) containing 2×GC buffer I (Takara-Bio), 200 µM deoxyribonucleotide triphosphate, 0.3 µM each of the universal primers, 0.625 U of LA *Taq* DNA polymerase (Takara-Bio), and template DNA. Amplification was performed with the following profile: 5 min at 94°C; 30 cycles of 1 min at 94°C, 3 min at 53°C, and 7 min of final extension at 53°C. PCR products were purified with the Wizard SV Gel and PCR Clean-up System (Promega, Tokyo, Japan), cloned into pGEM-T easy vector (Promega), and transformed into *ECOS* competent *E. coli* JM109 cells (NIPPONGENE, Tokyo, Japan). Using M13F and M13R primers, colony direct nucleotide sequencing was performed to the transformants. The sequence data were collected on an ABI3130 Genetic Analyzer, assembled, and analyzed by Bioedit (http://www.mbio.ncsu.edu/RNaseP/info/programs/BIOEDIT/bioedit.html).

**Table 1:** Sequences and melting temperature (*Tm*) of PCR primers used in this study.

PCR primers	Sequence	*Tm* (°C)
SP-1-5 (forward)^1^	CACACCGCCC GTCGCTACTA	70.3
ITS5 (forward)^2^	GGAAGTAAAAGTCGTAACAAGG	58.3
18SL (forward)	GGTGAACCTGCGGAAGGATCATTA	70.5
18S2 (forward)	AACCTGCGGAAGGATCATTA	63.2
5.8S (reverse)^3^	CGCTGCGTTCTTCATCG	64.5

1 Chu et al. [29],

2 White et al. [30],

3 Vilgalys and Hester [31].

### PNA-directed PCR clamping and diet analysis

PNA probes were designed based on the 18S rDNA and ITS1 sequences obtained in this study. Melting temperature (Tm) of PNA probe was estimated using a calculation tool (http://www.greiner-bio-one.co.jp/products/PNA/xls/PNA_Tm_OD.xls). To investigate the effects of PNA position and mismatches between PNA probes and the target sequence, PNA-directed PCR clamping was applied to four clones from two adult eels (eel00 and eel01) using 18SL and 5.8S primers under the same conditions mentioned above, with the addition of 4 µM of PNA probe. These four clones had 0 to three nucleotide mismatches with PNAs as described later. To confirm amplification, PCR products were electrophoresed on 1.5% (w/v) agarose gels followed by 20 min of staining with ethidium bromide.

For diet analysis, PNA-directed PCR clamping was applied to eight eel larvae using ITS5 and 5.8S primers under the same reaction conditions. When no apparent amplification of the eel ITS1 was observed, clamping was determined to be successful. The primary PCR products from the DNA templates extracted from eel larvae dorsal muscle and intestine were subjected to semi-nested second PCR using 18S2 and 5.8S primers. PCR products from the second PCR were cloned and sequenced as described above. ITS1 sequences obtained were subjected to homology search using a Basic Local Alignment Search Tool (BLAST) in GenBank. Because cross contamination from the larval body surface was unavoidable, we used following criteria for the interpretation: 1) sequences detected from the dorsal muscle alone or both dorsal muscle and intestine may not be from intestine, 2) sequences detected only from intestine may be from intestine, and 3) similar sequences shared by several larvae may be from prey candidates if the eel larvae are dependent upon certain organisms for food.

## Results

### Eel ITS1 sequence and design of PNA probes

**Figure 1:**
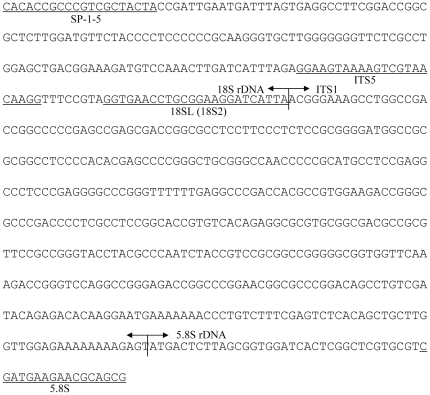
Nucleotide sequence of a 676 bp fragment from a common type clone (eel00-01) amplified from an adult Japanese eel (*Anguilla japonica*; eel00). This clone comprised 202 bp partial sequence of 18S rDNA, 422 bp entire sequence of ITS1, and 52 bp partial sequence of 5.8S rDNA. PCR primers are underlined.

**Figure 2:**
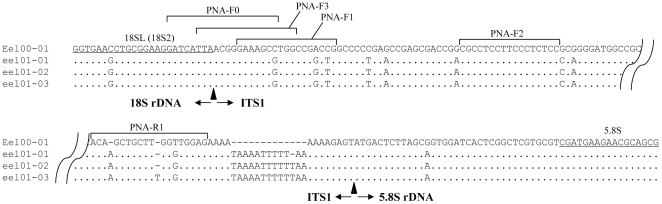
Sequence alignment among a common type clone (eel00-01) and three intra-individual variant clones (eel01-01 to -03). PCR primers (18SL and 5.8S) are underlined. The positions of five PNA probes (PNA-F0 to F3 and R1) are shown by a horizontal bracket. The sequences of all PNA but one (PNA-F3 having one mismatch) were fully complementary to those of eel00-01 (top sequence). PNA-F2 was fully complementary to all types. PNA-F0 and F3 were designed to have one and two substitutions against variant types, respectively. PNA-F1 and R1 were designed to have three substitutions against variant types.

The nucleotide sequence for a clone (eel00-1) obtained from an adult individual (eel00) is shown in Fig. 1, and has been deposited in the DNA Data Bank of Japan under accession No. of AB617806. This sequence is comprised of 202 bp partial sequence of 18S rDNA, 422 bp entire sequence of ITS1, and 52 bp partial sequence of 5.8S rDNA. The ITS1 region is characteristic of high GC content (69.9%) DNA. Nucleotide sequences of 100 clones obtained from the other three adults (eel01 to eel03, 30 to 40 clones each) were determined. Four to five ITS1 variants were observed in each individual, and 11 variants were determined in total. One variant was the most common and nearly identical to eel00-1, and the sequences of the other 10 variant types were deposited in the DNA Data Bank of Japan (AB617807 to AB617816). Cloning efficiency indicated that the variant types were minor intragenomic variants or pseudogenes. The length of ITS1 in these variant types were longer (431 to 437 bp) and the GC content less (60.7 to 61.4%) than those of the common type (eel00-1). The nucleotide sequence difference (all substitutions including indel were equally treated) between variant types and the common type was 16.2±1.7%, much larger than those within a variant type (1.8±0.4%) and within the common type (0.3±0.2%). Figure 2 shows the aligned nucleotide sequences of eel00-1 and three intra-individual variants from one adult (eel01); the flanking regions (18S rDNA-ITS1 and ITS1-5.8S rDNA) and positions of four PNA probes (PNA-F0 to F2 and R1) are designated. The nucleotide sequences and Tm of PNA probes are presented in Table 2. All PNA but one (PNA-F3) were a perfect match with the common type. PNA-F0 (19 bases) and PNA-F3 (17 bases), located in the flanking region between 18S rDNA and ITS1, shared eight and three nucleotides with the 18SL primer, respectively. PNA-F0 had one mismatch with the variant types. PNA-F3 (17 bases) had one mismatch with the common type and two with the variant types. PNA-F1 and PNA-R1 (17 and 18 bases, respectively) were designed to be near the 5′ end and 3′ end of ITS1, respectively, and had three mismatches, including gaps, with variant types. PNA-F2 was a perfect match with all types. The Tm of the PNA probes varied from 67.3°C (PNA-F2) to 86.7°C (PNA-F1).

**Table 2:** Sequences and melting temperature (*Tm*) of PNA probes used in this study.

PNA probes	Sequence	*Tm* (°C)
PNA-F0	GATCATTAACGGGAAAGCC	78.9
PNA-F1	GAAAGCCTGGCCGACCG	86.7
PNA-F2	CGCCTCCTTCCCTCTCC	67.3
PNA-F3	TTAATGGGAAAGCCTGG	77.5
PNA-R1	CTCCAACCAAGCAGCTGT	73.3

### PCR clamping efficiency in relation to mismatch and position of PNA

**Figure 3:**
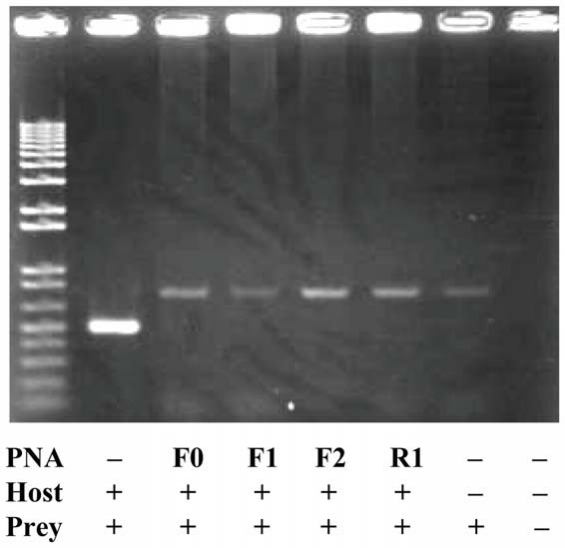
A result of PNA-directed PCR clamping using a mixed DNA template from a common type clone (eel00-01) and spiny lobster. Without PNA probe, eel ITS1 (c.a. 500 bp) was predominately amplified but lobster ITS1 was not (2^nd^ lane from the left). All PNA probes successfully inhibited amplification of eel ITS1 but did not inhibit amplification of lobster ITS1 (c.a. 680 bp; 3^rd^ to 6^th^ lane from the left).

**Figure 4:**
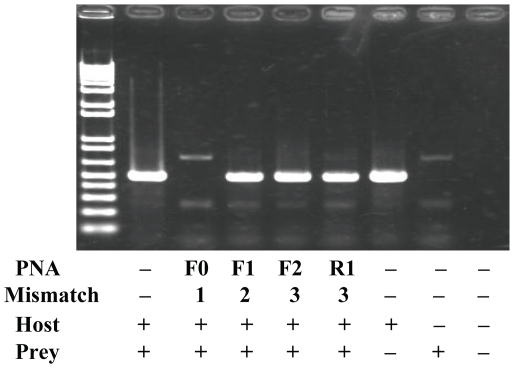
A result of PNA-directed PCR clamping using a mixed DNA template from a variant type clone (eel01-01) and spiny lobster. PNA-F0 (one mismatch) successfully inhibited amplification of eel ITS1 but did not inhibit amplification of lobster ITS1 (3^rd^ lane from the left), while the other PNAs (having two to three mismatches) failed to inhibit amplification of eel ITS1 (4^th^ to 6^th^ lane from the left).

The ITS1 regions of all template DNAs of the common type (eel00-1) and variant type clones (eel01-01 to eel01-03) of the Japanese eel and the Japanese spiny lobster were confirmed to be effectively amplified by PCR primer pairs (18SL and 5.8S). DNAs of the common type clone (eel00-1) and spiny lobster were mixed at a ratio of 100∶1, and the 100 pmol mixed DNA was used for PCR using the 18SL and 5.8S primers with and without eel-specific PNA probes. The result of the PCR clamping is shown in Fig. 3. Without PNA probe, eel ITS1 (c.a. 500 bp) was predominately amplified but lobster ITS1 was not (Fig. 3, 2^nd^ lane from the left). It appeared that the PCR favored dominant DNA molecules and shorter fragments for amplification. Amplification of the common eel ITS1 type (eel00-01) was successfully inhibited by all PNA probes (Fig. 3, 3^rd^ to 6^th^ lane from the left), although one PNA (PNA-F3) had one mismatch with the common type (not shown). When amplification of eel ITS1 was effectively inhibited, the lobster ITS1 (c.a. 680 bp) was amplified (Fig. 3, 3^rd^ to 6^th^ lane from the left). One clone (eel01-01) of the variant types was used to investigate the effect of mismatch between the PNA probes and template DNA (Fig. 4). PNA-F0 (one mismatch) successfully inhibited amplification of eel ITS1 but did not disturb amplification of lobster ITS1 (Fig. 4, 3^rd^ lane from the left), while eel ITS1 was predominately amplified with PNA-F3 (two mismatch), PNA-F1 (three mismatch), and PNA-R1 (three mismatch; Fig. 4, 4^th^ to 6^th^ lane from the left).

### Investigation of the natural diet of leptocephali

We used PNA-F0 to investigate the natural diet of the leptocephali. In all samples tested (dorsal muscle and intestine of eight larvae), no apparent amplification of the eel ITS1 was observed in the first and second rounds of PCR with PNA-F0. Forty-eight transformed colonies per sample were subjected to nucleotide sequence analysis using the M13 forward primer. By omitting non-ITS1 sequences and clones having no inserts, we obtained a total of 743 sequences (374 from dorsal muscle and 369 from intestine). Sequence alignments allowed us to classify the 743 sequences into 32 types (lep1 to 33; lep23 not included) and 49 sub-types (Table 3; GenBank accession no. AB616868-AB616909, AB616911-AB616920). We found very similar or nearly identical sequences in the database for 19 types (lep14 to 33), where the homology score between the query sequences and the highest similar sequences ranged from 85 to 99%. Among them, 17 types appeared to be fungi (lep16 to 33). The length of these fungal ITS1 sequences varied from 58 to 267 bp and GC content varied from 23.7 to 64.1%. Among fungal sequences, lep16 (*Malassezia* spp.) was detected in both dorsal and intestine samples of almost all larvae. One type (lep14) detected from dorsal muscle and intestine of a larva (St24-12) was identical to a macroalgal species (*Eckloniopsis radicosa*), and another (lep15) obtained from an intestine sample of a larva (St23-11) was identical to an undetermined eukaryote (AB490762). No other sequence identities were found in the database for the remaining 13 types (lep1 to 13), and these types were not related one another. According to the above-mentioned criteria, 12 types (lep2, 6, 8–10, 15, 18, 21, 24, 25, 27, and 30) may be from intestine and only one (lep21) was shared by different larvae.

**Table 3:** Assignments of ITS1 fragments detected from dorsal muscle (M) and intestine (S) of eight eel leptocephali.

Type	Sub	BLAST best hit	2009								2010								Length (bp)
	type*		163§		165		167		177		St23-10		St23-11		St24-12		St30-15		mean ± SD
			M	S	M	S	M	S	M	S	M	S	M	S	M	S	M	S	
lep1	4	none					29			22									234.6±0.9
lep2	1	none										10							391
lep3	2	none					2	5											231.5±4.9
lep4	1	none			7														172
lep5	1	none	1																154
lep6	1	none								11									136
lep7	1	none													1				151
lep8	1	none														23			142
lep9	1	none						1											149
lep10	1	none																9	329
lep11	1	none			3														91
lep12	1	none															4		217
lep13	1	none															7		381
lep14	1	*Eckloniopsis radicosa*													10	6			307
lep15	1	AB490762												3					362
lep16	5	*Malassezia* spp.	46	33	31	32	16	39	17	15	45	14	24		3	12	19	9	212.1±13.1
lep17	6	*Aspergillus* spp.				1			30						16	1		10	206.5±52.4
lep18	2	*Auricularia* sp.										13							155
lep19	2	*Cladosporium* spp.				7								7	12				153.5±0.6
lep20	3	*Candida* spp.		10															179.3±20.2
lep21	1	*Gibberella moniliformis*														4		5	151
lep22	1	*Rhodotorula mucilaginosa*			1	8													151
lep24	1	*Tinctoporellus epimiltinus*												3					178
lep25	1	*Articulospora* sp.												33					157
lep26	1	*Nigrospora* sp.											2						151
lep27	1	*Meyerozyma guilliermondii*																15	167
lep28	1	*Periconia* sp.											9						151
lep29	1	*Trametes trogii*											13						196
lep30	1	*Phlebia brevispora*										8							208
lep31	1	*Sporobolomyces salmoneus*															18		147
lep32	1	*Hyphodontia* sp.													4				177
lep33	1	*Yarrowia lipolytica*			4														58
		total	47	43	46	48	47	45	47	48	45	45	48	46	46	46	48	48	

*Nucleotide sequences of all sub-types are available in DNA database (AB616868-AB616909, AB616911-AB616920).

§ Larval strains named after sampling station.

## Discussion

### PNA directed PCR-clamping

All PNA probes successfully inhibited amplification of fully complementary eel ITS1, indicating that position of the PNA has little effect on clamping as long as the Tm is higher than that of the PCR primers. Two or more base pair mismatches between PNA probes and the template sequences were sufficient to dramatically reduce the clamping efficiency (Fig. 4), although designing PNA having higher Tm may improve clamping efficiency. A single mismatch is known to considerably decrease the Tm of PNA probe at 8–20°C [10]–[12]. Unsuccessful clamping using PNA-F3 and variant types indicated that the two bp mismatch lowered the Tm of the PNA below that of the primers. Igloi [12] using 11-mer PNA and its complementary DNA demonstrated the importance of the position of the DNA/PNA mismatch and mismatch types as well. Igloi [12] observed that a single mismatch positioned at the center or terminus of the PNA/DNA duplex was more stable than that positioned at 4 bases from terminus and that stabilization was maximum for G:T and T:T mismatched pairs and minimum for A:A and G:G pairs. In our study, PNA-F0 had one mismatch (C:C) with the variant types at terminus and PNA-F3 had one mismatch (G:T) with the common type at 5 bases from terminus. Both PNAs successfully inhibited amplification of variant and common types, respectively, suggesting that the Tm of these PNA were higher than that of the primers in spite of the presence of one mismatch.

Slight difference in amplification efficiency may be observed among PNAs used (see Fig. 3). Although the difference may be caused by loading amount and/or leakage of DNA samples in wells, non-specific PCR clamping can not be ruled out. PNA-F0 was designed in the flanking region between 18S rDNA and ITS1. The upper eight nucleotides are at 3′ end of 18S rDNA and universal, and subsequent three nucleotides are at 5′ end of ITS1 and semi-universal [6], which may elevate stabilization of the PNA on non-specific DNA templates.

### Investigation of natural diet of the leptocephali

The efficiency of PNA-directed PCR clamping adopted in the present study was remarkable, in that we found no clone containing eel ITS1 sequence among 743 clones examined. More than half of the sequence types obtained appeared to be of fungi, with the genus *Malassezia* extensively detected in our samples. This fungal genus comprises a group of superficial fungi occurring as skin flora on the human and animal body, but not in the environment [13]. Furthermore, only two (lep 19 and 28) among 17 fungal types detected are seen in the complete list of higher marine fungi (http://ocean.otr.usm.edu/~w529014/index_files/Page1195.htm). Therefore, many fungal strains detected in the present study may be the result of cross contamination during the handling process on the research vessel and/or in the laboratory. By omitting fungi, six types (lep 2, 6, 8–10, and 15) were thought to be from the intestine. No sequence similarity among them was observed, indicating that the leptocephali may not be dependent on a narrow range of organisms for their food source.

The variation in ITS1 length we observed ranged from 58 to 391 bp (Table 3). In contrast to the coding region of rDNA, large ITS1 length variations have been observed, even within closely related taxa. Variation in length of fungal ITS1 has been observed ranging from 140 to 1,100 bp [14], [15] and extreme length variation (791 to 2,572 bp) has been observed in ladybird beetles [16]. In marine animals, vertebrate ITS1 (Osteichthyes and Chondrichthyes) is relatively long (318 to 2,318 bp) compared with that of invertebrates (117 to 1,613 bp), and ITS1 of gelatinous animals (Cnidaria and Ctenophora) is especially short (118 to 422 bp) [17]. Amplification efficiency may be considerably different between shorter and longer fragments [18].

ITS1 sequences obtained in the present study were confined to the shorter range, suggesting that the detection of eukaryotes having shorter ITS1 was biased or that eel leptocephali consume eukaryotes having shorter ITS1. The results obtained in this study may correspond to a recent study of the diet of European eel (*A. anguilla*) larvae using 18S rDNA separated by denaturing gradient gel electrophoresis (DGGE) [19], which suggested that gelatinous zooplankton is common prey for the eel larvae. Riemann et al. [19] proposed that they detected a wide variety of animal phyla, including fungi and phytoplankton, from the larval eel gut; however, they did not analyze a control free from gut contents, such as dorsal muscle.

An alternative hypothesis is that eel leptocephali consume already-degraded material, which is consistent with information of Pfeiler [20] and Otake *et al.* [21] that eel leptocephali use dissolved organic matter and/or particulate organic matter (POM). Longer fragments may be more susceptible to digestion than shorter fragments [22]. On the other hand, oikopleurlid larvacean houses and zooplankton fecal pellets were observed in the gut of leptocephali of eight eel species, and were considered a major dietary component for leptocephalus larvae [23]. Furthermore, aloricate protozoa were considered to be a significant energy source for eel leptocephali [24]. However, wild-caught leptocephali of the conger eel *Conger myriaster* and cultured Japanese eel leptocephali were not attracted to *Oikopleura dioica* fed in the laboratory, even when the leptocephali contacted the larvacea (Dr. Kurogi, personal communication). Cultured leptocephali of the Japanese eel showed no active feeding behavior toward zooplankton [25]. Wild-caught leptocephali of the pike eel *Muraenesox cinereus* and conger eel *C. myriaster* were observed ingesting and defecating squid paste [26]. A slurry-type diet made from shark egg yolk powder was found to be a suitable food for captive-bred Japanese eel larvae [27], and the first cultivation of the glass eel in the laboratory was established by this diet supplemented with krill hydrolysate, soybean peptide, vitamins, and minerals [25]. Although the Japanese eel actively engulfs the slurry-type diet, they do so only when they contact the food (Dr. Masuda, personal communication). Otherwise, the food was unnoticed by leptocephali, indicating that, unlike other fish larvae, leptocephali do not seem to depend on visual and olfactory sensing for food. Based on his comprehensive review of leptocephalus feeding ecology, Miller [8] seemed fairly convinced that marine snow-like material is the major food for leptocephali. The present study detected no specific DNA common to leptocephalus larvae, and previous studies reported a wide variety of small organisms, larvacean houses, and fecal pellets as food [23], [24]. This corresponds to Miller's implication and is further corroborated by stable isotope ratio analysis on the eel leptocephali and POM [21], [28].
